# Early‐Life Secondhand Smoke Exposure and Development of Atopic Dermatitis up to Adulthood

**DOI:** 10.1111/cea.70204

**Published:** 2025-12-21

**Authors:** Anna Zettergren, Niklas Andersson, Anne‐Sophie Merritt, Inger Kull, Petter Ljungman, Erik Melén, Göran Pershagen, Susanne Lundin, Emma K. Johansson, Natalia Ballardini, Sandra Ekström, Anna Bergström

**Affiliations:** ^1^ Institute of Environmental Medicine, Karolinska Institutet Stockholm Sweden; ^2^ Centre for Occupational and Environmental Medicine Stockholm Sweden; ^3^ Department of Clinical Science and Education, Södersjukhuset Karolinska Institutet Stockholm Sweden; ^4^ Sachs' Children and Youth Hospital Stockholm Sweden; ^5^ Department of Cardiology, Danderyd Hospital Stockholm Sweden; ^6^ Department of Global Public Health Karolinska Institutet Stockholm Sweden; ^7^ Department of Medicine Solna Karolinska Institutet Stockholm Sweden

**Keywords:** atopic dermatitis, birth cohort, early‐life exposure, filaggrin, immunoglobin E, longitudinal, second‐hand smoke

**Abbreviations**
AD: Atopic dermatitis
BAMSE: Swedish acronym for Children Allergy Environment Stockholm Epidemiology (Barn Allergi Miljö Stockholm Epidemiologi)
CI: Confidence interval
*FLG*
: *Filaggrin*
GEE: Generalised estimating equations
IgE: Immunoglobin E
OR: Odds ratio
SHS: Second‐hand smoke

To the editor,

Early‐life secondhand smoke (SHS) exposure is suggested to increase AD risk, though previous findings are mixed and few have followed study participants to adulthood [1, 2, 3, 4]. Further, the association may differ depending on Immunoglobin E (IgE) sensitization [5] and mutations in the filaggrin gene (FLG) [6]. We aimed to study early‐life exposure to SHS and the development of AD up to adulthood in the Swedish prospective birth cohort BAMSE (*n* = 4089) [7]. We further aimed to investigate differences in the risk of AD in combination with IgE sensitization and in the presence of FLG mutations.

The study population included 3862 participants with data from the baseline questionnaire around age 2 months and at least 3 follow‐ups at ages 1, 2, 4, 8, 12, 16, and 24 years. Informed consent was obtained, and the study was approved by the Regional Ethics Committee at Karolinska Institutet in Stockholm, Sweden.

At baseline, maternal smoking during pregnancy and current parental smoking was assessed through questionnaires. AD was assessed through questionnaires at each follow‐up and defined based on having dry skin in combination with an itchy rash on typical locations and/or doctor's diagnosis. IgE sensitization was assessed from serum at ages 4, 8, 16, and 24 years (*n* = 3476). FLG mutations (in any of del4, R2447X, R501X) were assessed from serum collected at 8 or 16 years (*n* = 2747).

Early‐life SHS exposure was explored as (1) exposure to maternal smoking during pregnancy or (2) exposure to smoking by either or both parents during infancy. Overall and age‐specific associations between early‐life SHS exposure and development of AD from ages 1–24 years were assessed by logistic regression using generalised estimating equations (GEE). Main models were adjusted for the presence of older siblings, family socioeconomic status, parental allergic disease, and the presence of dampness or mould in baseline household. Models for parental smoking during infancy were additionally adjusted for maternal smoking during pregnancy and exclusive breastfeeding duration. Additionally, to assess the singular effect of each exposure window, sensitivity analyses were performed excluding participants exposed to the other exposure window.

Further, multinomial logistic regression using GEE was used to assess the association between early‐life SHS and AD in combination with IgE sensitization up to 24 years as, (1) no AD and no IgE sensitization, (2) IgE sensitization without AD, (3) AD without IgE sensitization and (4) AD and IgE sensitization. Lastly, interactions between early‐life SHS exposure and FLG mutations were assessed by logistic regression using GEE with an interaction term between the exposure variable and FLG mutations. For details on methods, see https://zenodo.org/records/17864630.

There was no statistically significant overall association between maternal smoking during pregnancy and AD up to age 24 years, and no clear pattern for age‐specific associations (Figure 1). However, after exclusion of participants exposed to parental smoking during infancy (*n* = 795), an overall association with AD was suggested (OR: 1.30, 95% CI: 0.98, 1.73), and significant associations were observed at ages 12 (OR: 1.84, 95% CI: 1.15, 2.95) and 24 years (OR: 1.61, 95% CI: 1.03, 2.53). Moreover, the strength of the association increased with age (*p* for trend = 0.049). Parental smoking during infancy was associated with an overall increased risk of AD up to 24 years (OR: 1.25, 95% CI: 1.07, 1.47) and at ages 8 (OR: 1.34, 95% CI: 1.06, 1.70), 12 (OR: 1.36, 95% CI: 1.05, 1.77), 16 (OR: 1.54, 95% CI: 1.15, 2.07), and 24 years (OR: 1.41, 95% CI: 1.11, 1.78), but the association did not significantly increase with age (*p* for trend = 0.183) (Figure 1). After exclusion of participants exposed to maternal smoking during pregnancy (*n* = 491), the association appeared stronger, both overall (OR: 1.36, 95% CI: 1.14, 1.62) and for most age‐specific associations.

**FIGURE 1:**
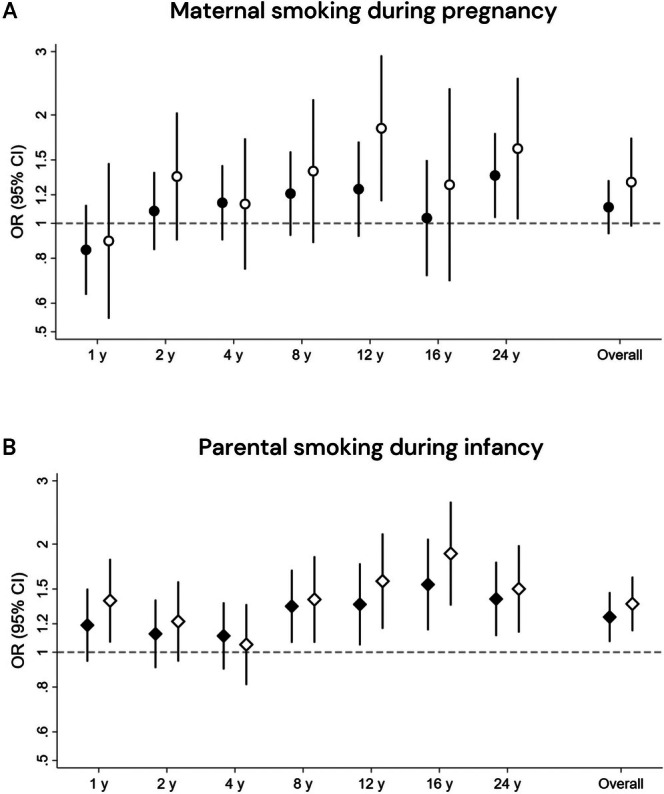
Assocations between early‐life secondhand smoke exposure and atopic dermatitits prevalence from age 1–24 years for maternal smoking during pregnancy (A) and parental smoking during infancy (B). Filled circles: Main results from logistic regression using generalised estimating equations adjusted for sex, presence of older siblings, family socioeconomic status, parental allergic disease, presence of dampness or mould in household, exclusive breastfeeding duration (only B) and maternal smoking during pregnancy (only B). Filled symbols: Main model. Empty symbols: Main models excluding participants exposed to parental smoking during infancy (*n* = 795) (A)/maternal smoking during pregnancy (*n* = 491) (B).

Age trajectories of AD were additionally explored, and maternal smoking during pregnancy was associated with both persistent AD (OR: 1.60, 95% CI: 1.14, 2.23) and late onset AD (OR: 1.79, 95% CI: 1.02, 3.14), while parental smoking during infancy was associated with persistent AD only (OR: 1.48, 95% CI: 1.08, 2.02). No associations were found for early transient or intermittent AD (https://zenodo.org/records/17864630).

Analyses of AD in combination with IgE sensitization showed that parental smoking during infancy was associated with an overall increased risk of AD with IgE sensitization from 1 to 24 years (OR: 1.39, 95% CI: 1.14, 1.70), but not with AD without IgE sensitization. In contrast, maternal smoking during pregnancy was not associated with any phenotype. For details, see (https://zenodo.org/records/17864630).

No interactions were found between FLG mutations and early‐life SHS exposure on the risk of AD up to 24 years (*p* = 0.512 for maternal smoking during pregnancy, *p* = 0.369 for parental smoking during infancy).

In this longitudinal birth cohort study, both maternal smoking during pregnancy and parental smoking during infancy were independently associated with an increased risk of developing AD up to young adulthood. For parental smoking during infancy, the risk was particularly increased for AD in combination with IgE sensitization, which is in line with previous findings [8, 9]. Our findings support previous studies [3, 4], but differ from several studies with shorter follow‐up time and differences in exposure assessment [1, 2].

We found no evidence of an interaction between early‐life SHS exposure and FLG mutations on AD risk up to adulthood; however, these analyses have limited power and should be interpreted with caution.

To conclude, early‐life exposure to SHS was associated with increased risk of persistent AD up to young adulthood. The risk was higher for AD in combination with IgE sensitization.

## Author Contributions

A.Z.: conceptualization, methodology, validation, formal analysis, writing, original draft, writing, review and editing, visualisation. N.A.: methodology, data curation, writing, review and editing. A‐S.M.: resources, writing, review and editing. I.K.: investigation, resources, writing, review and editing. P.L.: conceptualization, supervision, writing, reviewing and editing. E.M.: resources, supervision, writing, reviewing and editing. G.P.: writing, reviewing and editing. S.L.: writing, reviewing and editing. E.K.J.: writing, reviewing and editing. N.B.: writing, reviewing and editing. S.E.: conceptualization, data curation, methodology, supervision, writing, reviewing and editing. A.B.: conceptualization, methodology, project administration, resources, funding acquisition, supervision, writing, original draft.

## Funding

This work was supported by Vetenskapsrådet (2016‐03086, 2018‐02524, 2020‐02170). Forskningsrådet om Hälsa, Arbetsliv och Välfärd (2017‐00526). Svenska Forskningsrådet Formas (2016‐01646). Hjärt‐Lungfonden, Astma‐ och Allergiförbundet, Region Stockholm, Naturvårdsverket (NV‐09284‐13, NV‐00175‐15) Karolinska Institutet.

## Conflicts of Interest

The authors declare no conflicts of interest.

## Data Availability

Data not publicly available, Link to supplementary repository: https://zenodo.org/records/17864630.
